# MOFs Containing Solid‐State Electrolytes for Batteries

**DOI:** 10.1002/advs.202206887

**Published:** 2023-01-22

**Authors:** Shu Jiang, Tingting Lv, Yi Peng, Huan Pang

**Affiliations:** ^1^ Interdisciplinary Materials Research Center, Institute for Advanced Study Chengdu University Chengdu 610106 P. R. China; ^2^ School of Chemistry and Chemical Engineering Yangzhou University Yangzhou Jiangsu 225009 P. R. China

**Keywords:** composites, metal–organic frameworks, solid‐state batteries, solid‐state electrolytes

## Abstract

The use of metal–organic frameworks (MOFs) in solid‐state electrolytes (SSEs) has been a very attractive research area that has received widespread attention in the modern world. SSEs can be divided into different types, some of which can be combined with MOFs to improve the electrochemical performance of the batteries by taking advantage of the high surface area and high porosity of MOFs. However, it also faces many serious problems and challenges. In this review, different types of SSEs are classified and the changes in these electrolytes after the addition of MOFs are described. Afterward, these SSEs with MOFs attached are introduced for different types of battery applications and the effects of these SSEs combined with MOFs on the electrochemical performance of the cells are described. Finally, some challenges faced by MOFs materials in batteries applications are presented, then some solutions to the problems and development expectations of MOFs are given.

## Graphical abstract

The use of metal–organic frameworks (MOFs) in solid‐state electrolytes (SSEs) has received widespread attention in the modern world. SSEs can be divided into different types, some of which can be combined with MOFs to improve the electrochemical performance of the battery by taking advantage of the high surface area and high porosity of MOFs.

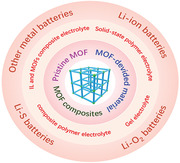

## 1 Introduction

With the development of society and the growth of population, human demand for energy has been increasing.^[^ ^1^ ^]^ In recent decades, some renewable clean energy sources, such as solar energy and wind energy, have been gradually developed, but their energy conversion efficiency is not satisfactory.^[^ ^2^ ^]^ Thus, to meet the energy needs of industrial production or daily life, people need energy storage equipment that is safe, durable, clean, and convenient.^[^ ^3^ ^]^

As one of the most frequently used energy storage devices, batteries are commonly applied in various electronic devices. In order to pursue higher energy density, lithium batteries have become the main direction of people's research. Due to their extremely high theoretical energy density (3860 Wh kg^−1^), less weight, smaller size, reduced environmental impact, and other benefits, lithium batteries are used in a broad variety of commercial products, including electric vehicles and mobile phones.^[^ ^4^ ^]^ Lithium‐ion batteries generally use liquid electrolytes (LEs). In fact, concentration gradients and cell polarization originating from the opposite transport of lithium ions may lead to uneven deposition of lithium ions in the repeated charge and discharge process. This could result in the growth of lithium dendrites, which could cause short circuits, decreased capacitance, leakage, explosions, and other safety incidents. To solve these problems, the researchers tried a variety of approaches: Wang et al. prepared graphene thin films to strengthen the lithium metal anode, preventing the electrode from bending to produce lithium dendrites.^[^ ^5^ ^]^ Sun et al. attempted to prepare an Al–Li alloy cathode that inhibited the growth of lithium dendrite.^[^ ^6^ ^]^ However, these measures do not really solve the existing problems with lithium batteries.

Researchers from Mac discovered that the solid‐state electrolytes (SSEs) of LiAsF_6_ structure inhibited the formation of lithium dendrites, causing further research to be conducted. Solid‐state batteries (SSBs) represent a novel approach. SSBs are impermeable and resistant to high temperatures. They are composed of solid‐state electrodes and SSEs, of which SSEs have excellent stability.^[^ ^7^ ^]^ Most importantly, the SSEs can inhibit the generation of lithium dendrites, reduce the occurrence of battery short circuits, improve the stability of the battery. Most importantly, the SSEs can inhibit the formation of lithium dendrites and improve the stability of lithium batteries. For example, Liu et al. developed a structural framework of garnet structures as 3D ion skeletons.^[^ ^8^ ^]^ In addition, the SSEs are also used in Zn batteries: a self‐standing gelatin‐based SSE was reported by Han et al. and it inhibits corrosion and passivation of Zn batteries and improves the cycle stability of batteries.^[^ ^9^ ^]^ However, ordinary SSEs also have many areas to be improved, such as the improvement of ion conductivity, the consistency of the electrode–electrolyte interface contact, etc. Later, Hurd et al. found that the electrolytes added to the metal–organic frameworks (MOFs) had better electrochemical properties.^[^ ^10^ ^]^ Gerbaldi et al. significantly improved the ionic conductivity of poly(ethylene oxide) composite electrolyte membranes by using Al‐based MOFs as fillers, while Shen et al. developed quasi‐solid‐state electrolytes with bionic ion channels by exploiting the multimetal open‐site property of MOFs, Liu et al. synthesized MOFs complexes by modifying MOFs with polycaprolactone in order to improve the conductivity of pristine solid‐state polymer electrolytes (SPEs), which successfully improved the cyclability and stability of lithium‐ion batteries. Long et al. attempted to synthesize MOFs derivatives linked with Fe‐Ni‐LDH to form high‐performance catalysts, which significantly improved the electrochemical performance of Zn–air batteries.^[^ ^11^ ^]^ Consequently, MOFs became increasingly valuable to researchers.

MOFs composed of metal nodes and organic linkers, which can form a variety of unique structures, so they are widely used in the preparation of various energy storage equipment materials.^[^ ^1^, ^12^ ^]^ MOFs have the following benefits: first, they can be in full contact with other components due to high porosity, large specific surface area, excellent kinetics, and great reactivity.^[^ ^4^, ^7^, ^13^ ^]^ MOFs have high surface polarity, which permits them to control the Lewis acid–base interaction in the system, thus improving their electrochemical properties.^[^ ^7^, ^14^ ^]^ Some by‐reaction products and impurities can be adsorbed due to high surface energy, which are caused by high porosity, high specific surface area and other characteristics.^[^ ^14^, ^15^ ^]^ Second, MOFs can be adapted to different materials due to variety of structures, which make MOFs easily composite with different electrolyte materials and use their excellent electrochemical properties to modify SSEs. It can improve the ionic conductivity, ion migration, and other electrochemical properties of SSEs. Third, MOFs can also form MOF‐derived materials by pyrolysis or ion‐assisted solvothermal conversion processes. Besides these, the pore size and pore type of MOFs can be freely controlled. In this way, MOFs can form a special ion screen to promote the transfer of cations and improve the uniformity of ion migration.^[^ ^1^, ^14^, ^16^ ^]^

**Scheme 1:**
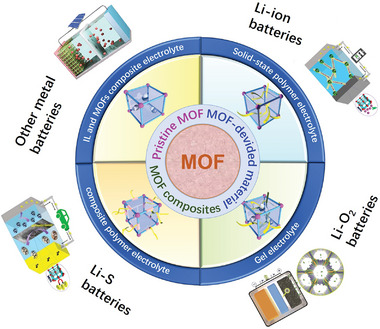
Illustration of MOFs composites for SSEs. Reproduced with permission.^[^ ^19^ ^]^ Copyright 2021, The Royal Society of Chemistry. Reproduced with permission.^[^ ^18^ ^]^ Copyright 2022, Elsevier. Reproduced with permission.^[^ ^18^ ^]^ Copyright 2020, Wiley‐VCH.

Herein, we classify different types of SSEs and describe the changes in these electrolytes after the addition of MOFs. After that, they are described in different batteries, where the SSEs of the MOFs are attached to the modification of the electrochemical properties of various batteries. Finally, some challenges of MOFs materials in battery applications are proposed (**Scheme** **1**).

## 2 Solid‐State Electrolytes Containing MOFs

**Scheme 2:**
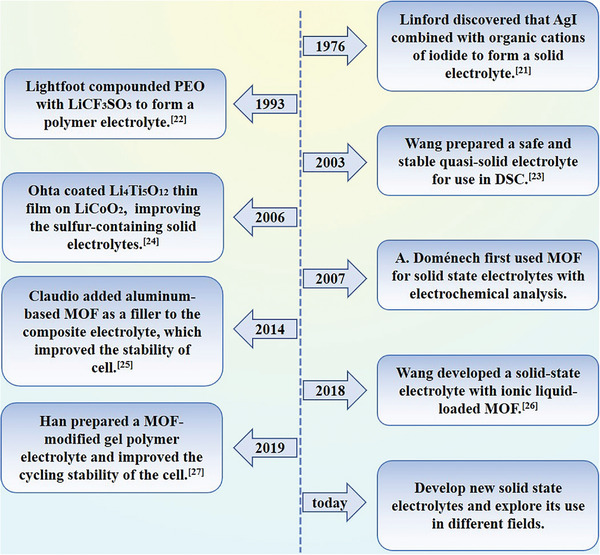
A succinct account of the evolution of SSEs: the efficient contribution in 1976,^[^ ^20^ ^]^ 1993,^[^ ^21^ ^]^ 2003,^[^ ^22^ ^]^ 2006,^[^ ^23^ ^]^ 2007, 2014,^[^ ^11a^ ^]^ 2018,^[^ ^24^ ^]^ 2019,^[^ ^25^ ^]^ and development prospect of SSEs.

SSEs can be divided into the following two categories based on the amount of liquid electrolytes: all‐solid‐state electrolytes (the content of the liquid electrolytes is 0%), and quasi‐solid‐state electrolytes (the content of the liquid electrolytes <5%).^[^ ^17^ ^]^ All‐solid‐state electrolytes can be divided into inorganic solid‐state electrolytes, SPEs, and composite polymer electrolytes (CPEs). Quasi‐solid‐state electrolytes are also known as gel polymer electrolytes (GPEs).^[^ ^18^ ^]^ **Scheme** **2** illustrates the most significant radical transformation of the SSEs. In addition, there are some essential concerns about the application of MOFs that need to be taken into account. First, MOFs should have channels that facilitate ion transport, because the lower ionic conductivity as well as the electrochemical window tends to limit the use of SSEs, and it is meaningless if the MOFs cannot provide similar channels. Second, SSEs with MOFs attached should have sufficient Young's modulus, whereby only sufficient mechanical strength can prevent the growth of dendrites. Third, MOFs attachments need to maintain stability in contact with the electrode interface, unstable cycling may lead to inhomogeneous deposition of ions. In general, the addition of MOFs to SSE will enhance the strength of its polymer matrix to a certain extent. This will make its ion channels more stable, thereby further increasing the rate of ion transfer. Next, classifications will be described in detail.^[^ ^19^ ^]^

### 2.1 Solid Polymer Electrolytes

The SPEs offer many advantages, such as low preparation cost, mechanical flexibility, good processability, compatibility with electrodes, which make it the ideal electrolytes for SSBs.^[^ ^11^, ^26^ ^]^ However, the poor mechanical strength and ionic conductivity limit their application.^[^ ^27^ ^]^ These structures may lead to the growth of lithium dendrites. Therefore, people enhance it by in situ cross‐linking or combining with other materials to inhibit the growth of lithium dendrites, thereby enhancing its mechanical softness and improving ionic conductivity.^[^ ^28^ ^]^ Moreover, nonsatisfactory ion transport capacity as well as particle agglomeration caused by inorganic nanofillers at high concentrations also reduce ionic conductivity and prevent its further development. The porous structure of MOFs solves this problem well.^[^ ^11b^ ^]^ In addition, it was surprising to find that MOFs consist of various central metal ions and organic ligands, where the central metal ions can act as active sites for ion transport and thus facilitate ion conduction. Moreover, MOFs can inhibit the appearance of lithium dendrites due to their mechanical strength.

**Figure 1:**
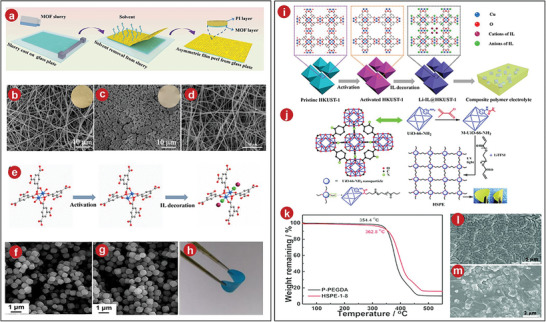
a) The PI‐ZIF8 film preparation scheme. b,c) Modified and unmodified SEM images of the PI film's ZIF‐8 layer. d) SEM of pristine PI film. e) Procedure for Li‐IL@HKUST‐1. f) SEM of HKUST‐1. g) SEM images of Li‐IL@HKUST‐1. h) Elastic CPEs membrane. i) Synthesis of CPEs integrated with Li‐IL@HKUST‐1. j) The hybrid covalently connected MOFs‐PEGDA‐based all‐solid‐state electrolytes synthetic method. k) TGA curves of P‐PPEGDA and HSPE‐1‐8. l) Surface of HSPE‐1‐8. m) Cross‐section of HSPE‐1‐8. a–d) Reproduced with permission.^[^ ^29^ ^]^ Copyright 2020, Wiley‐VCH. e–i) Reproduced with permission.^[^ ^34^ ^]^ Copyright 2020, American Chemical Society. j–m) Reproduced with permission.^[^ ^35^ ^]^ Copyright 2018, The Royal Society of Chemistry.

For example, MOFs consisting of flexible polymer chains covalently linked to flexible independently mixed all‐solid polymer electrolytes films were successfully synthesized (**Figure** **1**a),^[^ ^29^ ^]^ in which the two sides of the SPEs film were modified and unmodified morphologies (Figure 1b,c), the unmodified part was similar to the original PI film (Figure 1d), while there were many MOFs nanoparticles in the modified test part. In such asymmetrically designed SPEs, the MOFs layer suppressed the appearance of Li dendrites and improved the smoothness of Li^+^ transport, greatly enhancing the safety and stability of lithium batteries.

### 2.2 Composite Polymer Electrolytes

The electrochemical performance of SPEs is ultimately limited. It cannot meet the performance requirements of all batteries. However, CPEs, which combines the advantages of various materials, are excellent solutions.^[^ ^11^, ^30^ ^]^ CPEs are typically composed of a variety of materials, including MOFs particles, polymer matrices, and lithium salts.^[^ ^19^, ^31^ ^]^ In general, commonly used polymers include polyethylene oxide (PEO), polyvinylidene fluoride (PVDF), polystyrene sulfonate, polyvinyl alcohol (PVA), etc., which can be combined with MOFs with special diameter and pore shape to form a series of MOFs composites. It is not only that these composite substances have all the advantages of MOFs, such as stability, plasticity, more active metal sites, high porosity, and so on, but they also display Lewis acid–base interactions and relying on the transfer route provided by MOFs particles, greatly promote ion conduction and polymer motion segments, thereby improving the electrochemical performance of the batteries.^[^ ^14^, ^32^ ^]^ Therefore, as excellent composite materials, CPEs composited with MOFs have excellent prospects as SSEs.^[^ ^14^, ^33^ ^]^

As common electrolytes, polyethylene oxide PEO‐based electrolytes have high utilization and practical performance, but their ionic conductivity at ambient temperature is not high. In order to improve its shortcomings, Wang et al. added MOFs into electrolytes to prepare composite polymer electrolytes.^[^ ^34^ ^]^ Wang et al. interacted ionic liquid with 1,3,5‐benzenetricarboxylic acid and copper complexes (HKUST‐1) to form MOFs complexes, dispersed it into the precursor solution of PEO‐based polymer, and then formed CPEs film by solution casting method. (The process is shown in Figure 1i, and the mechanism is shown in Figure 1e.) The surface of the composite CPEs film was very smooth and tough (Figure 1h). The characterization by scanning electron microscope (SEM) and transmission electron microscope (TEM) indicated that the structure of HKUST‐1 remained basically unchanged after the action of Li^+^ liquid (Figure 1f,g). In addition, the ionic conductivity of the synthesized CPEs film at 30 °C approached 1.20 × 10^−4^ S cm^−1^, which was far superior to the original PEO.

To improve ionic conductivity and optimize battery performance, the target product UiO‐66NH_2_‐PEGDA (vinyl functionalized MOF with poly(ethylene glycol) diacrylate) was synthesized by Wang et al. (Figure 1j).^[^ ^35^ ^]^ From the SEM characterization results, MOFs particles were uniformly attached to the target matrix, indicating that the composite results were favorable (Figure 1l,m). In addition, the CPEs film can maintain stability in high temperatures (Figure 1k) with a certain toughness and flexibility.

**Figure 2:**
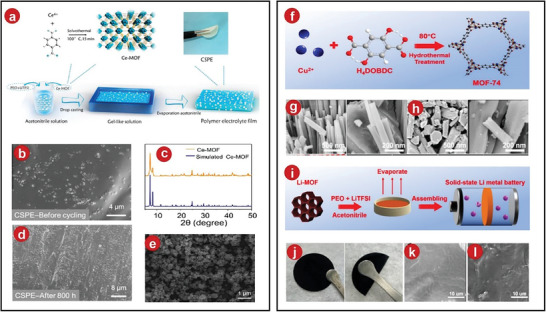
a) The production of Ce‐MOFs and Ce‐MOFs‐based composite SPEs film was illustrated schematically. b) Precycling SEM images of a CSPE membrane. c) Ce‐MOFs and synthetic Ce‐MOFs in XRD. d) Postcycling SEM images of a CSPE membrane. e) SEM of Ce‐MOFs nanoparticles. f) Cu‐MOFs‐74 structure was shown schematically. g) SEM of MOFs. h) SEM of Li‐MOFs. i) Li‐MOFs/PEO fabrication techniques and solid‐state Li–metal battery assembly methods. j) Image of the Li‐MOFs/PEO film. k) SEM of PEO. l) SEM of Li‐MOFs/PEO. a–e) Reproduced with permission.^[^ ^36^ ^]^ Copyright 2021, Elsevier. f–l) Reproduced with permission.^[^ ^37^ ^]^ Copyright 2022, American Chemical Society.

Besides, Wu et al. reported a Ce‐based MOF with abundant catalytically active sites, namely, Ce‐based metal–organic framework (Ce‐MOF), and added this species as a nanofiller to PEO‐based electrolytes to improve its Li^+^ conductance rate and the stability of the electrolytes.^[^ ^36^ ^]^ The specific process was shown in **Figure** **2**a. There were many grains on its surface, with a certain phenomenon of grain aggregation as evidenced by the SEM and XRD image (Figure 2c,e). After preparing the CPEs films, the result showed that the film maintains its original flexibility and was not easily broken. In addition, after compounding with Ce‐MOFs, the surface of the CPEs film did not change significantly after a certain period of cycling, which also proved its excellent stability (Figure 2b,d).

Recently, the lithium ion mobility was improved by coordinating the anions on the metal sites.^[^ ^37^ ^]^ This method was proposed by Zhang et al., they prepared Cu‐MOFs‐74 (Cu^2+^ with 2,5‐dioxido‐1,4‐benzenedicarboxylate) particles by a hydrothermal method, and utilized their characteristic of abundant Cu^2+^ sites to coordinate with bis(trifluoromethylsulfonyl)amide (TFSI), which can immobilize anions, and release free Li^+^, thereby improving ionic conductivity and battery cycling efficiency stability. The process of preparing MOFs was shown in Figure 2f. From SEM images, it can be observed that the MOFs particles exhibit a rod‐like morphology and a polygonal cross‐section (Figure 2g,h). The process of synthesizing CPEs and assembling the battery was shown in Figure 2i, the synthesized Li‐MOFs/PEO has excellent foldability and recovery ability (Figure 2j).The surface of uncompounded PEO was very rough. There were some cracks due to the high crystallinity, while the composite PEO did not have this phenomenon due to the decrease in crystallinity (Figure 2k,l).

**Figure 3:**
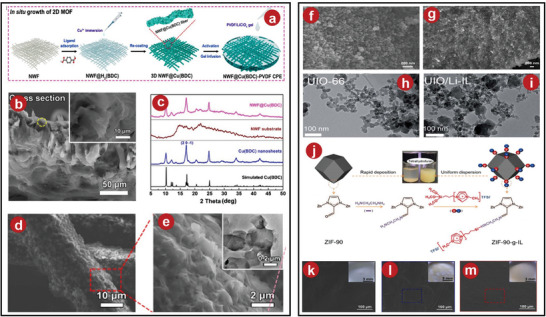
a) Diagram illustrating the fabrication steps for the NWF@Cu(BDC)PVDF CPEs. b) XRD patterns of Cu(BDC) samples. c) Cross‐sectional morphology of the NWF@Cu(BDC)‐PVDF CPEs as seen in SEM image. d,e) SEM of NWF@Cu(BDC). f) SEM of the MOFs at its initial state. g) Li‐IL@MOFs pellet cross‐sectional as seen by SEM. h) UIO‐66 in TEM images before absorbing Li‐IL. i) UIO‐66 in TEM images after absorbing Li‐IL. j) Schematic of ZIF‐90‐g‐IL. k) LiTFSI‐equipped SPEs at PEO as seen in SEM. l) LiTFSI‐equipped SPEs at PEO/ZIF‐90 as seen in SEM. m) LiTFSI‐equipped SPEs at PEO/ZIF‐90‐g‐IL as seen in SEM. a–e) Reproduced with permission.^[^ ^38^ ^]^ Copyright 2021, American Chemical Society. f–g): Reproduced with permission.^[^ ^24^ ^]^ Copyright 2018, Wiley‐VCH. h,i) Reproduced with permission.^[^ ^43^ ^]^ Copyright 2019, The Royal Society of Chemistry. j–m) Reproduced with permission.^[^ ^44^ ^]^ Copyright 2021, Elsevier.

To demonstrate the feasibility of engineering fast Li^+^ conduction, Yu et al. developed a new type of CPEs by exploiting the characteristics of 2D‐copper p‐dibenzoate (Cu(BDC)) MOFs with more open metal sites.^[^ ^38^ ^]^ The CPEs were enhanced by this MOFs scaffold, improving Li^+^ conductivity, and enabling it to achieve stable cycling in solid‐state Li batteries. The exact synthesis procedure was displayed (**Figure** **3**a). The XRD analysis results of this MOFs sample were shown in Figure 3b, which were consistent with the expected synthesis. SEM revealed that the Cu(BDC) sample formed a tight network structure (Figure 3c). The SEM scan of the composite CPEs product revealed that the surface morphology of the sample was very smooth (Figure 3d), with the MOFs‐based scaffold completely embedded in the PVDF matrix, whose internal 3D network structure greatly promoted the filler and polymer. The interfacial interaction provided a fast transport path for Li^+^.

### 2.3 Ionic Liquid and MOFs Composite Electrolytes

Although the SSEs loaded with MOFs have excellent performance, they still have some minor flaws, such as the relatively large interfacial resistance of MOFs. In order to further improve the electrochemical performance of these composite electrolytes, people try to start from MOFs and further combine it with other materials to make up for these shortcomings.^[^ ^39^ ^]^ Traditional organic solvents can no longer meet people's needs because of their volatile, flammable, and low ionic conductivity. Fortunately, ionic liquid (IL) has been found to possess many attractive properties compared to conventional organic solvents, including high stability, incombustibility, low volatility, and high ionic conductivity. Inspired by this, a composite material of IL and MOFs was prepared and named IL@MOFs.^[^ ^40^ ^]^ In general, several methods are commonly used to prepare IL@MOFs, including postimpregnation: where MOFs are synthesized and then impregnated with IL. Ship‐in‐bottle method: the precursor of IL is first put into the pore of MOFs, and then the precursor is converted into IL. Capillary method: IL and MOFs are mixed with mortar, and then the mixture is annealed to evenly disperse IL into the pores of MOFs.^[^ ^41^ ^]^ The commonly used IL are generally Li^+^‐containing liquids (Li‐IL), which lose their original liquid characteristics after being loaded into the MOFs lattice. Unlike conventional SSEs, it does not have a solid–solid interface, but a nanowetted interface instead, which reduces the interfacial resistance, and the open channels in the MOFs can also put it in contact with the electrodes, thereby promoting Li^+^ transport and improving the thermal and mechanical stability of the battery.^[^ ^14^, ^42^ ^]^

Consider solving interface problems and improving battery electrochemical performance, a good idea was presented by Wang et al.^[^ ^24^ ^]^ The composite electrolytes were successfully prepared by [EMIM_0.8_Li_0.2_][TFSI] and MOFs‐525(Cu) (a highly porous open framework constructed by Zr_6_O_4_(OH)_4_ clusters and [5,10,15,20‐tetrakis(4‐carboxyphenyl)porphyrin]Cu(II) organic linkers), (Figure 3f,g). The obtained MOFs crystal was found to be basically spherical after scanning by SEM, and the battery composed of these electrolytes had a unique nanointerface wetting effect, which greatly improved the performance of the battery.

Later, Wu et al. designed the C_48_H_28_O_32_Zr_6_ (UIO‐66) as MOFs and made it composited with Li‐IL as a filler, which not only improved the ionic conductivity and suppressed lithium dendrites, but also improved its stability by mixing with PEO.^[^ ^43^ ^]^ They used a solvothermal method to prepare monodisperse nanocrystals, then mixed and heated with Li‐IL to successfully prepare University of Oslo (UIO)/Li‐IL composite fillers (Figure 3h,i). The crystal structures were almost identical, but the specific surface area of the latter was reduced. Because Li^+^ can move freely in the nanochannel, it had excellent lithium‐ion conductivity.

To ensure that MOFs fillers were distributed uniformly throughout the polymer matrix, Lei et al. tried an IL to graft MOFs fillers.^[^ ^44^ ^]^ They prepared ZIF‐90 as the MOFs material, then dehydrated and condensed the IL containing siloxane groups to prepare ZIF‐90‐g‐IL. The process was shown in Figure 3j. The obtained product was characterized, and it was found that the distribution of the nanofiller in the PEO‐based SPEs was more uniform than that of the original PEO‐based SPEs, which improved its uniformity (Figure 3k–m), thereby further improving the effective performance of the SPEs.

### 2.4 Gel Polymer Electrolytes

Although traditional SSEs have many advantages, they also have some unavoidable disadvantages, such as scaled processability and large electrode interface resistance. At this time, GPEs were proposed.^[^ ^45^ ^]^ Compared with solid electrolytes, GPEs have better plasticity and lower interfacial resistance. Compared with liquid electrolytes, GPEs can inhibit the formation of lithium dendrites and are safer and more stable.^[^ ^1^, ^46^ ^]^ In general, common GPEs contain polymers, lithium salts, and organic plasticizers. Common polymers include PEO, polypropylene oxide, polyacrylonitrile (PAN), and polyvinylidene fluoride hexafluoropropylene (PVDF‐HFP), which have high ionic conductance and can be used as substrates for GPEs.^[^ ^47^ ^]^ However, some organic nonconductive fillers may not be able to balance ionic conductivity and compatibility between electrodes and interfaces.^[^ ^48^ ^]^ Therefore, some attempts have been made to introduce MOFs into GPEs to make use of the high ionic conductivity of MOFs materials and the stability of the interface with electrodes.^[^ ^49^ ^]^

**Figure 4:**
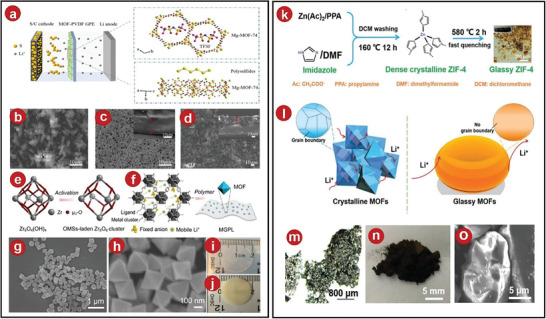
a) MOFs–PVDF GPEs schematic with anions immobilized for the Li–S battery. b–d) SEM images of Mg‐MOFs‐74, PVDF film, and MOFs–PVDF film. e) Principle of OMS preparation. f) Mechanism of Li^+^ conduction. g,h) SEM images of UiO‐66. i,j) Physical photos of GPL film and MGPL film. k) Diagram of the glassy ZIF‐4 preparation process. l) Figure of the conductivity of Li^+^ in crystalline and glassy MOFs. m) Glass ZIF‐4 block picture from optical microscope. n) Photo of glassy ZIF‐4 after grinding. o) SEM image of glassy ZIF‐4 after grinding. a–d) Reproduced with permission.^[^ ^25^ ^]^ Copyright 2019, American Chemical Society. e–j) Reproduced with permission.^[^ ^50^ ^]^ Copyright 2020, American Chemical Society. k–o) Reproduced with permission.^[^ ^51^ ^]^ Copyright 2021, Wiley‐VCH.

Considering safety, Han et al. synthesized Mg‐MOFs‐74 and added it to enhance PVDF‐based GPEs in order to stabilize the lithium anode.^[^ ^25^ ^]^ They prepared MOFs‐modified PVDF GPEs by vacuum filtration (**Figure** **4**a). The original MOF exhibited a rod‐like structure, which was uniformly attached to the PVDF membrane after synthesis (Figure 4b–d). After testing, although the resistivity of the modified PVDF was higher than before, the ionic conductivity was significantly improved due to the increase in thickness. In addition, due to the porous nature of MOFs, it absorbed the electrolytes better and the battery can cycle stably.

In order to improve Li^+^ mobility, a Zr‐based MOF named as UiO‐66 by Lu was used as a microporous filler for GPEs. It contained a large amount of Lewis acid OMS and can immobilize anions and enhance Li^+^ mobility (Figure 4e,f).^[^ ^50^ ^]^ UiO‐66 was synthesized as octahedra‐like particles with excellent thermal stability (Figure 4g,h). The original colorless and transparent GPEs film turned pale yellow after adding MOFs (Figure 4i,j), and with the increase of MOFs loading, the surface roughness and porosity increased.

Recently, Jiang et al. fabricated the GPEs for lithium–metal batteries by ZIF‐4 glass in order to solve the problem of abundant grain boundaries in traditional MOFs.^[^ ^51^ ^]^ Due to the isotropic nature of ZIF‐4 glass and the absence of grain boundaries, the ion transport was very uniform (Figure 4l), which suppressed the appearance of lithium dendrites. The preparation process was shown in Figure 4k. The obtained glassy ZIF‐4 was ground into brown transparent particles, and the SEM images showed irregular particles (Figure 4m–o). In addition, this type of battery also had excellent cyclability and had broad application prospects.

## 3 Application of MOFs in Li‐Based Batteries

As current mainstream batteries, Li‐based batteries have excellent prospects in electric vehicles and electronic products.^[^ ^52^ ^]^ The commonly used lithium‐based batteries are liquid lithium‐ion batteries, which have the advantages of repeatable charge and discharge and high electric capacity. Generally, organic liquid is utilized as electrolytes, graphite carbon materials are utilized as negative electrode, lithium salts as positive electrodes, and charge and discharge are carried out by ion deintercalation mechanism.^[^ ^53^ ^]^ With the development of traditional liquid lithium‐ion batteries, more problems follow: the original battery capacity is no longer adequate to meet people with the increasing needs of them. Lithium ions can be transported unevenly, resulting in the formation of lithium dendrites piercing the separator due to the difference in concentration gradient and battery polarization. It can also cause battery short circuit, leakage, or even explosion and other harmful consequences. In order to better solve these problems such as the insufficient capacitance and the formation of lithium dendrites, many attempts are tried, such as electrolytes additives, electrode protection, and separator modification. As the lithium is stripped from the properties of the electrolytes during the charging and discharging process, cracks will appear in the interphase of SSEs. This will result in side reactions between liquid electrolytes and lithium metal. Therefore, in a liquid electrolytes system, these methods cannot completely solve the problem of lithium dendrite.^[^ ^29^, ^54^ ^]^ As time goes by, people attempt to introduce SSEs to improve their contact with electrodes, thus preparing solid‐state lithium batteries due to advantages of being nonvolatile, nonflammable, high mechanical flexibility, reasonable safety, etc. However, it also has poor ion transport ability. In order to improve these shortcomings, people attach much importance to the excellent properties of MOFs materials such as high plasticity and more metal active sites, and introduce MOFs into solid‐state lithium batteries to further improve their electrochemical performance.^[^ ^55^ ^]^

### 3.1 Li‐Ion Batteries

#### 3.1.1 Working Mechanism

Lithium‐ion batteries (LIBs) have been widely used in a variety of mobile electronic devices and large machinery as the mainstream battery today.^[^ ^56^ ^]^ LIBs have the advantages of high temperature performance, large capacity, no memory effect, light weight, and environmental protection.^[^ ^53^, ^57^ ^]^ During charging and discharging, Li^+^ migrates to the negative or positive electrode through the polymer diaphragm. However, the unmodified Li electrode obtains a sharp and porous Li metal structure during the initial stripping process, which results in the formation of Li dendrites in the subsequent Li plating process.^[^ ^56^, ^58^ ^]^ The introduction of MOFs can improve this situation.^[^ ^14^, ^24^, ^59^ ^]^ There are two main mechanisms by which MOFs work to improve Li‐ion batteries. The first one is construction of ion sieves. The introduction of MOFs with appropriate pore size and strong cation sites in SSEs can restrict the migration of anions. The high specific surface area of MOFs can also promote sufficient contact between Li^+^ and other components, reduce the activation energy and interfacial resistance required for transport, and thus enable uniform Li^+^ flux distribution, resulting in uniform Li^+^ electrodeposition. The pore size of MOFs can also play the role of screening. In the process of preparing MOFs, the reaction conditions can be controlled to obtain MOFs with suitable pore size, which can play a screening role for different anions, thus improving the conductivity of Li^+^ and enhancing the electrochemical performance of the batteries. The other is immobilization of anions. MOFs have independent framework structures, and these frameworks can immobilize the anions in the electrolyte very effectively by electrostatic or adsorption effects, creating stable and efficient Li^+^ channels that allow Li^+^ to move freely. In general, a common method of immobilizing anions is to bind the anion to a neutral unsaturated metal site. In Li‐ion batteries, by immobilizing the anion, it is also possible to successfully dissociate the anion from the Li^+^, thus reducing the crystallinity of the electrolyte and enabling rapid migration of the Li^+^. In addition, the addition of some MOFs complexes and MOFs derivatives can also broaden the electrochemical window of the electrolyte and improve the stability of the battery cycle.

#### 3.1.2 Application

**Figure 5:**
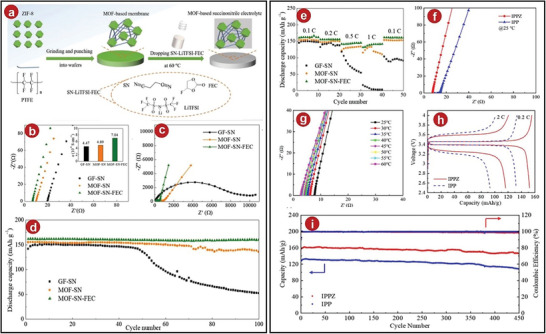
a) An illustration showing how the MOFs‐SN‐FEC electrolyte was made. b) EIS evaluations of the electrolytes achieved at room temperature. c) EIS curves after 100 cycles. d) Cellular cycling stability with all provided electrolytes at 0.1 C. e) Test the cells' functioning at various rates. f) IPPZ and IPP Nyquist graphs at 25 °C. g) Nyquist plots of IPPZ from 25 to 60 °C. h) Battery voltage curves at 0.2 and 2 C for the LFP/IPPZ/Li and LFP/IPP/Li batteries. i) Battery performance comparison between LFP/IPPZ/Li and LFP/IPP during cycling at 0.2 C. a–e) Reproduced with permission.^[^ ^60^ ^]^ Copyright 2021, American Chemical Society. f–i) Reproduced with permission.^[^ ^61^ ^]^ Copyright 2022, American Chemical Society.

Among all the solutions to the easy formation of Li dendrites in Li batteries, the construction of ionic sieves for modulation is a novel and interesting approach. Recently, Han et al. synthesized an MOFs‐based succinonitrile electrolyte by high‐temperature impregnation method (**Figure** **5**a) and named it MOFs‐SN‐FEC.^[^ ^60^ ^]^ Using the voids of MOFs to act as an ion sieve in the GPEs, this not only limited the transfer of other bulk ions, accelerated the transfer of Li^+^, but also made the transfer of Li^+^ more uniform, avoiding the generation of lithium dendrites due to uneven transmission. At the same time, the lithium‐ion conductivity test of the prepared electrolytes was carried out. It found that the result was 7.04 × 10^−4^ S cm^−1^, and it was much superior to that of the uncomposed MOFs material (Figure 5b). In addition, the relevant electrochemical performance of the MOFs‐SN‐FEC electrolytes was tested in an LIB. According to the result, the battery still had a capacity retention rate of 98.8% after 100 cycles at 0.1 C (Figure 5d). The interface resistance and charge transfer resistance were 368 Ω (Figure 5c), such a small resistance also proved the excellent stability of the LIB using MOFs‐SN‐FEC. Besides these, the charge–discharge ability of the battery was tested (Figure 5e). The battery had excellent rate performance at different rates. In addition, there was no deposition of lithium dendrites during the process. This further proved the safety and stability of the battery.

GPEs have relatively flexible texture, which can reduce the interfacial resistance between the electrolytes and electrodes. Qi et al. prepared a C_8_H_12_N_4_.Zn (ZIF‐8)/PP composite film by the situ growth method, and composited with Li‐ion liquid to synthesize GPEs, which was named IPPZ.^[^ ^61^ ^]^ The ionic conductivity of the IPPZ GPEs was tested at 25 °C. It was discovered that it can reach 2.09 × 10^−4^ S cm^−1^, a value that was significantly greater than that of other SSEs. At the same time, the ionic conductivity increased with the temperature. It also illustrated the excellent ionic conductivity of the IPPZ GPEs (Figure 5f,g). The LIB was assembled using the IPPZ GPEs, and its electrochemical performance was tested. The battery was found to have an excellent initial discharge capacity of 167.9 mAh g^−1^. Even after 450 cycles at 0.2 C, the capacity was still more than 90% of the original (Figure 5i), which was more stable and less polarized (Figure 5h). It also showed that the type of electrolytes had excellent application prospects in LIBs.

**Figure 6:**
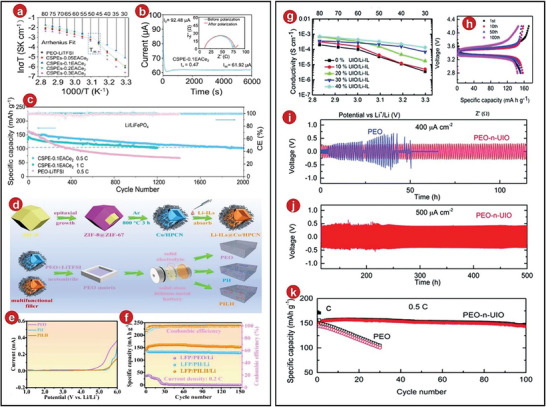
a) CSPEs’ Arrhenius graphs based on various EACe_2_ concentrations and their linear fitting in various temperature ranges using the Arrhenius equation. b) Li^+^ transference number of CSPE‐0.1EACe_2_ was measured. c) Li/PEO‐LiTFSI/LiFePO_4_ cell and Li/CSPE‐0.1EACe_2_/LiFePO_4_ cell cycling performance. d) Diagram of the synthesis process and assembly strategy of solid‐state lithium batteries. e) Electrochemical stability window of CSEs. f) Battery cycling results. g) Temperature‐dependent conductivity of the PEO‐n‐UIO SSEs. h) Cell potential versus particular capacity plot. i,j) Voltage distributions in the Li/PEO‐n‐UIO/Li cell at 60 °C. k) Particular battery capacities with galvanostatic charge/discharge cycling at a rate of 0.5 C at 60 °C. a–c) Reproduced with permission.^[^ ^62^ ^]^ Copyright 2021, Elsevier. d–f) Reproduced with permission.^[^ ^63^ ^]^ Copyright 2021, Elsevier. g–k) Reproduced with permission.^[^ ^43^ ^]^ Copyright 2019, The Royal Society of Chemistry.

Infinite coordination polymers (ICPs) have grain structures with well‐defined geometries and thus have better shape control capabilities. This inspiration has enlightened Wu et al., they designed a Ce‐EA polyphenol ICP (EACe_2_) with a highly amorphous structure and composited it with SPEs to prepare a CSPE‐EACe_2_ composite electrolytes.^[^ ^62^ ^]^ In the process of testing ionic conductivity, the composite content ratio of CSPE and EACe_2_ was 10:1 (CSPE‐0.1EACe_2_). The composite electrolytes at this time had the highest ionic conductivity, which was 2.76 × 10^−4^ S cm^−1^ at 30 °C (**Figure** **6**a), and this result was also much higher than other uncombined electrolytes. The Li^+^ migration number at this time was 0.47 (Figure 6b). The electrolytes also performed very well in LIBs. After 5 cycles, the interfacial resistance between CSPE‐0.1EACe_2_ composite electrolytes and LiFePO_4_ was 20.5 Ω cm^2^, and it was very stable afterward. This value was also smaller than the interfacial resistance between other electrolytes and LiFeO_4_. The initial discharge capacity of this cell was 161.3 mAh g^−1^, and after 2000 cycles at 0.5 C, it still had 63% of its original capacity (Figure 6c).

Among all measures to modify MOFs materials, the introduction of IL is a good solution. The hollow structure and high surface area of MOFs can effectively absorb IL, which can improve electrochemical properties such as ionic conductivity. Zhang et al. took these advantages into account, then they synthesized novel SSEs from Co‐doped MOFs derivatives, Li‐IL, PEO polymer matrix, and other materials (Figure 6d).^[^ ^63^ ^]^ The ionic conductivity of the electrolytes at 30 °C was ≈1.91 × 10^−4^ S cm^−1^, and the ionic conductivity could be further improved when the temperature increased. The electrochemical stability window was also consistently lower than 4.2 V during the oxidation process, which was smaller than that of the other comparison groups (Figure 6e). Applying it to LIB, it still had excellent cycle performance: after 150 cycles at 0.2 C, the battery still had a discharge specific capacity of 152.9 mAh g^−1^ (Figure 6f), and no formation of lithium dendrites also illustrated the feasibility of MOFs‐based derivative composites in lithium SSBs.

MOFs materials are generally composed of central metal ions and organic ligands. They are very effective nanoporous materials. The internal void structure is also very suitable for accommodating other particles to form MOFs derivatives. Besides having the original advantages, this method also improves the disadvantages of the original MOFs material. Here, Wu et al. selected UIO‐66 as the MOFs material and made it absorb Li‐ion liquid by solvothermal method to prepare a MOFs‐derived nanoporous material, then named it UIO/Li‐IL.^[^ ^43^ ^]^ At 30 °C, the SSEs mixed with 40% UIO/Li‐IL showed a 37‐fold increase in conductivity compared to the initial PEO electrolytes, reaching 1.3 × 10^−4^ S cm^−1^. (The conductivity of the composite polymer electrolytes calculated from AC impedance spectroscopy is shown in Figure 6g.) In addition, the nanostructured UIO/Li‐IL filler improved the stability for LIBs compared to the initial PEO matrix: the battery operated well at a current density of 500 at 60 °C (Figure 6i,j), while the original PEO‐based electrolytes became unstable only at 400 due to the infiltration of lithium dendrites. In addition, the specific capacity of the battery for charge and discharge was 151 and 170 mAh g^−1^, respectively, then the coulombic efficiency was 89% at the rate of 0.5 C. However, in the following cycles, the coulombic efficiency was greatly improved, reaching 99%. The specific discharge capacity of the cell was still very stable, even after 100 cycles, it remained at 95% (Figure 6h–k).

**Figure 7:**
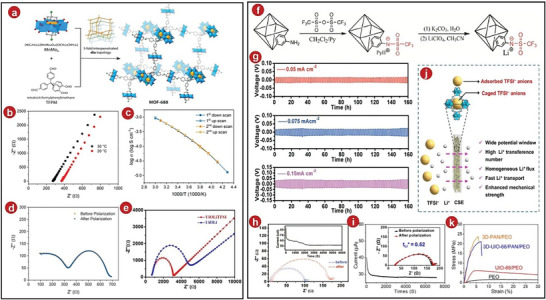
a) Mechanism of MOFs‐688 synthesis. b) Nyquist plots of MOFs‐688 at 20 and 30 °C. c) Temperature as a function of ionic conductivity. d) Nyquist plots of the Li|MOFs‐688|Li cell before and after polarization. e) UiOLi and UiOLiTFSI at 25 °C EIS graphs. f) The synthesis process of UiOLiTFSI single Li‐ion conducting SSEs. g) At various current densities, the PL/UiOLiTFSI exhibits Li plating–stripping behavior. h) EIS for the Li|PL/UiOLiTFSI|Li cell before and after polarization. i) 3D‐UIO‐66/PAN/PEO/LiTFSI SSEs. j) Ion transfer via a networked 3D‐UIO‐66/PAN/PEO/LiTFSI CSE was shown schematically. k) PEO/LiTFSI, 3D‐PAN/PEO/LiTFSI, UIO‐66/PEO/LiTFSI, and 3D‐UIO‐66/PAN/PEO/LiTFSI SSE stress–strain curves. a–d) Reproduced with permission.^[^ ^64^ ^]^ Copyright 2019, American Chemical Society. e–g) Reproduced with permission.^[^ ^58b^ ^]^ Copyright 2019, American Chemical Society. j–k) Reproduced with permission.^[^ ^65^ ^]^ Copyright 2022, Elsevier.

Later, an intrinsically anionic framework was prepared by Xu et al., then they named it MOFs‐688 (the procedure was shown in **Figure** **7**a).^[^ ^64^ ^]^ They tested the Li^+^ exchange conductivity of MOFs‐688 by EIS. After multiple cycles at −40 to 60 °C, they found that at 20 and 30 °C, its ionic conductivity was 3.4 × 10^−4^ and 4.6 × 10^−4^ S cm^−1^ (Figure 7b,c). Due to the special structures of the MOFs, most of the anions of the metal salts were fixed on the metal framework. This result was much better than the Li^+^ migration number of the liquid electrolytes. And it was found by EIS that there was a stable interface between MOFs and lithium metal. The resistance of the interface was further tested, which was 353.2 and 353.3 Ω, respectively (Figure 7d). The resistance was lower than other SSEs, reflecting the excellent electrochemical performance of the MOFs‐688 after compounding.

Recently, It was modified by Zhu et al. to produce an anionic MOFs‐based single‐ion conducting electrolytes which was called Li/University of Oslo Li bis(trifluoromethylsulfonyl)amide (UiOLiTFSI) after its synthesis (Figure 7f).^[^ ^58b^ ^]^ It was calculated that the ionic conductivity of Li/UiOLiTFSI was 2.07 × 10^−4^ S cm^−1^ at 25 °C, the result was far superior than other SSEs (Figure 7e). Zhu et al. believed that the immobilized anions in UiOLiTFSI can separate the ion pairs of LiTFSI, which can increase the concentration of lithium ions and further improve the ionic conductivity. In addition, the activation energy of Li/UiOLiTFSI was 0.31 eV, it was much lower than that of other electrolytes, so the electrolytes can be considered as a superelectronic conductor. The Li^+^ migration number was tested by EIS in LIBs, and the outcome of these electrolytes was discovered to be 0.84 (Figure 7h), which was higher than other materials. In addition, the LIB was tested with constant current cycle (Figure 7g). Due to its excellent interfacial stability, it was very stable at room temperature and a current density of 0.05 mA cm^−2^, and the average polarization voltage was also lower than 0.02 V. Therefore, the battery can avoid polarization to a certain extent, and has excellent prospects in fast‐charging solid‐state lithium batteries.

Solid composite electrolytes, such as those used in typical batteries, can improve the electrochemical performance of the battery to a large extent, but generally speaking, they cannot enhance the Li^+^ conductivity sufficiently in the battery. The 3D interconnected MOFs network is a relatively novel design. The 3D interconnected MOFs network can not only provide an efficient path for the transport of Li^+^ and promote the uniform and fast passage of Li^+^, but also limit the transport of other anions. Additionally, it can increase the mechanical strength of the CPEs as well as improve its stability and safety. A 3D interconnected MOFs‐derived SSE, 3D‐UIO‐66/PAN/PEO/LiTFSI SSE, was fabricated and tested electrochemically in LIBs by Li et al.^[^ ^65^ ^]^ Its Li^+^ migration number was tested to be 0.52 (Figure 7i), which was better than other types of SSEs. By investigating its mechanism, it can be found that the pore size and adsorption capacity of UIO‐66 effectively limited the transport of anions, and the 3D network provided a pathway for the transport of Li^+^ (Figure 7j). The Young's modulus of the 3D MOFs network structure was very high, which was 819.4 MPa (Figure 7k), indicating that it had excellent mechanical strength. In the LIBs, the electrochemical performance of the electrolytes was also very stable, and there was no abnormality after 700 h of cycling.

### 3.2 Li–S Batteries

#### 3.2.1 Working Mechanism

Compared with other lithium‐based batteries, Li–S batteries have higher energy density, low cost, less self‐discharge phenomenon, and higher conversion efficiency of battery energy.^[^ ^66^ ^]^ Nonetheless, Li–S batteries also have some disadvantages that cannot be ignored: the electronic conductivity of sulfur is poor, Li–S batteries will generate some macromolecular lithium polysulfides (LiPSs) intermediates during cycling.^[^ ^67^ ^]^ They will shuttle back and forth between the ends of the electrode and deposit on the surface of the electrode. In order to try to solve these problems, researchers tried to introduce Li–S batteries with SSEs, which can effectively prevent the dissolution of LiPSs intermediates in them and prevent their shuttle effect. In addition, Li–S batteries can also be further optimized by taking advantage of the unique structures and functions of MOFs materials.^[^ ^68^ ^]^ To improve the electrochemical performance of Li–S batteries, starting from the electrodes is an effective approach. On the one hand, high performance sulfur‐based cathodes are tried. Such substrates generally need to have a high physical or chemical adsorption of sulfur so that LiPSs generated during cycling are effectively trapped and thus the shuttle effect is suppressed. In general, MOFs materials are excellent matrix materials by virtue of their pore structure and the ability to freely select their particle size. The pore size of MOFs is also an important factor. A larger pore size can load more sulfur, and a smaller pore size can inhibit the shuttling of polymorphs. Experimentally, it was found that a particle size of ≈200 nm could maintain efficient trapping ability of LiPSs, which suppressed the shuttle effect. Although the cell conductivity is not ideal due to the effect of solid MOFs, this can be solved by compounding MOFs with other high conductivity materials to form MOFs complexes with excellent conductivity and MOFs derivatives. On the other hand, a suitable electrolyte can also be prepared to stabilize the lithium anode of Li–S batteries. The theoretical electrical capacity of lithium anode is high, but it gradually degrades during cycling due to repeated plating and stripping, the shuttle effect of LiPSs also affects the stability of the solid‐electrolyte interface. The SSEs have the advantage of smaller size compared with the traditional liquid electrolyte, which can free up more space for the anode and thus increase the energy density of the battery. The electrolytes compounded with MOFs have higher ion selectivity, and the metal ions in MOFs can also provide many unsaturated coordination sites for Lewis bases, thus promoting Lewis acid–base interaction, and due to the effect of spatial site resistance, MOFs can lock the bulk anions and reduce their influence on the electrolyte, regulating the transport of lithium ions and the corresponding anions. Then they can avoid the appearance of space charge region, thus inhibiting the growth of lithium dendrites and improving the stability of lithium anodes, then improving the electrochemical performance of Li–S batteries.

#### 3.2.2 Application

**Figure 8:**
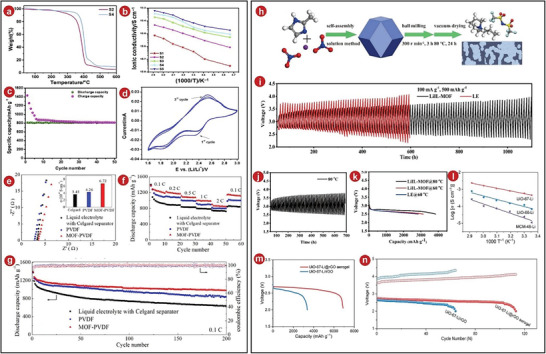
a) Thermograms of various CPEs from 25 to 600 °C. b) Arrhenius plot of the ionic conductivity as a function of the inverse temperature. c) Cyclic voltammetry profiles of the lab‐scale all solid‐state Li–S polymer cell. d) Constant current cycle diagram of the all‐solid‐state Li/S_4_/SGM cell. e) The relevant ionic conductivity at room temperature and the impedance spectra of the cells with varied electrolytes. f) Ability of the cells with various electrolytes at varied rates. g) The capacity of the cells with various electrolytes to cycle at a rate of 0.1 C. h) Schematic diagram of the steps involved in synthesizing a quasi‐solid LiIL‐MOFs electrolytes. i) Performance of the Li–O_2_ battery during cycling using LiIL‐MOFs at 60 °C. j) Performance of the Li–O_2_ battery during cycling using LiIL‐MOFs at 80 °C. k) Curves of Li–O_2_ batteries with LE and LiIL‐MOFs for deep discharge. l) Ionic conductivity of different SSEs as shown by the corresponding Arrhenius plot. m) At a current density of 100 mA g^−1^, the full discharge curves of Li–O_2_ batteries using different aerogel. n) The cycle life of Li–O_2_ batteries made of different aerogel with a current density of 100 mA g^−1^. a–d) Reproduced with permission.^[^ ^69^ ^]^ Copyright 2018, Elsevier. e–g) Reproduced with permission.^[^ ^25^ ^]^ Copyright 2019, American Chemical Society. h–k) Reproduced with permission.^[^ ^72^ ^]^ Copyright 2022, American Chemical Society. l–n) Reproduced with permission.^[^ ^73^ ^]^ Copyright 2022, Wiley‐VCH.

Recently, Shruti et al. synthesized aluminum terephthalic acid metal–organic frameworks (Al‐TPA‐MOFs) by an electrolytic method. They designed MOFs as additive for composite polymer electrolytes of Li–S batteries. The composite electrolytes prepared in this way had high thermal stability and did not decompose until 270 °C (**Figure** **8**a).^[^ ^69^ ^]^ Analyses of ionic conductivity of the electrolytes were conducted and found that when the composite amount of Al‐TPA‐MOFs was 10%, the ionic conductivity was the highest (Figure 8b), and it was assembled into an all‐solid‐state Li–S polymer battery. The cycle curve was tested (Figure 8c). After the first cycle, the positions and characteristic intensities of the oxidation/reduction peaks in the figure did not change much, indicating that the Li–S battery had an excellent cycling stability. In testing the discharge capacity of the battery, its capacity exceeded 800 mAh g^−1^ during the first discharge (Figure 8d). The MOFs prevented the dissolution of polysulfides, thereby inhibiting the migration of polysulfides to the electrodes, further improving the efficiency of the cells.

When the GPEs are applied in Li–S batteries, it can not only reduce the probability of lithium dendrite formation, but also improve the energy density of the battery. Han et al. selected Mg‐MOFs‐74 as the MOFs material.^[^ ^25^ ^]^ After composite modification with PVDF‐based GPEs, the performance and life of quasi‐solid‐state Li–S batteries were greatly improved. They tested the ionic conductivity of the resulting composite electrolytes and found that the result of the electrolytes composited by MOFs was 6.72 × 10^−4^, which was larger than uncombined one (Figure 8e). The ionic conductivity was also tested, and it was found that the electrolytes material after MOFs composite was as high as 0.66, it was higher than the electrolytes without MOFs composite, indicating that MOFs had the property of immobilizing anions here. The battery containing MOFs–PVDF composite electrolytes had lower polarizability and reversibility, it showed good cycling performance during the cycling process of Li–S battery. Each time the capacity fade was less than 0.15% (Figure 8g). In addition, it also had excellent cycle performance. At different rates, its discharge capacity was very high, especially at a rate of 2.0 C, the discharge capacity reached 860.5 mAh g^−1^ (Figure 8f). The result was much better than other GPEs batteries.

### 3.3 Li–O_2_ Batteries

#### 3.3.1 Working Mechanism

Li–O_2_ batteries have very high theoretical energy density and are potential choices as a modern electric vehicle battery.^[^ ^3^, ^53^, ^70^ ^]^ Conventional Li–O_2_ batteries are generally liquid electrolytes, but the safety, stability, and electrochemical performance of liquid electrolytes are not ideal, these prevent the further commercialization. However, these problems can be improved with the introduction of SSEs.^[^ ^71^ ^]^ Of course, ordinary SSEs have certain limitations on the development of Li–O_2_ batteries due to their large interfacial resistance and poor mechanical strength. Therefore, researchers have further modified the electrochemical performance of Li–O_2_ batteries by trying to introduce MOFs materials or prepare GPEs. In Li–O_2_ batteries, SSEs containing MOFs are more often utilized as the cathode to stable batteries. The MOFs material can provide a medium for electron transfer, and its porous nature can also allow sufficient diffusion of O_2_ and protect the cathode from moisture and CO_2_. The SSEs formed by MOFs can also form a good contact interface with the cathode, overcoming the point‐to‐point contact between the Li^+^ conductor and the e^−^ conductor, expanding the effective contact area of the cathode. The contact interface between anode, electrolyte, and cathode is also optimized, which reduces the interfacial resistance and polarization rate and maintains the cycling stability of Li–O_2_ batteries. In addition, some of the MOFs‐derived materials are able to act as catalysts to facilitate the redox reactions.

#### 3.3.2 Application

Due to the stability and good electrochemical performance of IL at high temperatures, IL and ZIF‐67 materials were composited in GPEs by Liu and it was called as LiIL‐MOFs (Figure 8h).^[^ ^72^ ^]^ The electrolytes had excellent ionic conductivity due to the conduction path provided by the ZIF‐67 material, which reached 4.14 × 10^−3^ S cm^−1^ at 80 °C. Next, they assembled a Li–O_2_ battery containing LiIL‐MOFs and found that it had excellent cycling performance: At a current density of 100 mA g^−1^, the LiIL‐MOFs Li–O_2_ battery remained stable after 100 cycles, while ordinary Li–O_2_ battery with the liquid electrolytes of 100% failed completely after less than 60 cycles (Figure 8i). And after testing, the LiIL‐MOFs Li–O_2_ battery can be stably cycled for more than 1000 h. In addition, Liu et al. also tested the cycling of the LiIL‐MOFs Li–O_2_ battery at high temperature. They found that the battery also had 70 effective cycles at 80 °C (Figure 8j), and the capacitance of the battery can be further improved with increasing temperature (Figure 8k), which also illustrated the feasibility of this LiIL‐MOFs Li–O_2_ battery at high temperature.

Later, a new solid‐state Li–O_2_ battery was designed and reported by Wang et al.^[^ ^73^ ^]^ They prepared Li‐ion‐conducted C_84_H_52_O_32_Zr_6_@reduced graphene oxide (UiO‐67‐Li@rGO) solid‐state cathodes using UiO‐67‐Li MOFs materials as SSEs and introducing MOFs on the surface of reduced graphite oxide aerogel on the cathode side. Subsequently, the ion migration number of the electrolytes was tested: at an applied voltage of 10 mV, the solid‐state battery with UiO‐67‐Li composite had an ionic conductivity of 0.64 mS cm^−1^, which was higher than that of other SSEs (Figure 8l), showing the MOFs material has excellent potential in promoting Li^+^ transport. Besides, the introduced rGO aerogel structure can enhance the O_2_ transfer and generate a large amount of TPBs, thus significantly improving the commercial value of Li–O_2_ batteries. After testing, this solid‐state battery was discovered to have a greater discharge capacity (Figure 8m). In addition, the solid‐state battery of UiO‐67‐Li@rGO cycled for 115 cycles before the voltage dropped to 2 V, and the capacity was fixed at 500 mAh g^−1^ (Figure 8n), which was significantly higher than other Li–O_2_ batteries. According to this result, it further illustrated the stability of this Li–O_2_ battery.

## 4 Electrochemical Application of MOFs in Other Batteries

### 4.1 Zn‐Based Batteries

#### 4.1.1 Working Mechanism

Compared with Li‐based batteries, Zn‐based batteries have the advantages of lower price, better safety performance, and less pollution.^[^ ^74^ ^]^ However, in practical use, they have disadvantages such as low electron transfer rate and poor cycle performance, which limit the further application of Zn‐based batteries.^[^ ^75^ ^]^ The main role of SSEs containing MOFs is to immobilize the anions, which have many pores and can be adsorbed and trapped by Lewis acid–base action, thus creating a channel for the smooth transport of Zn ions. These SSEs are also very compatible with Zn anodes. A special nanowetting interface can be generated between them, which can effectively optimize the deposition of Zn and make Zn behave smoothly and densely during the deposition process, thus avoiding the generated Zn dendrites from piercing the diaphragm and causing a short circuit in the cell. In addition, these MOFs can also serve as excellent cathode catalysts in Zn–air batteries, providing transport channels for oxygen reduction reaction (ORR) and oxygen evolution reaction (OER) through their porosity, facilitating the transport of intermediates, improving the efficiency of ORR and OER, and thus enhancing the electrochemical performance of the cells.

#### 4.1.2 Application

**Figure 9:**
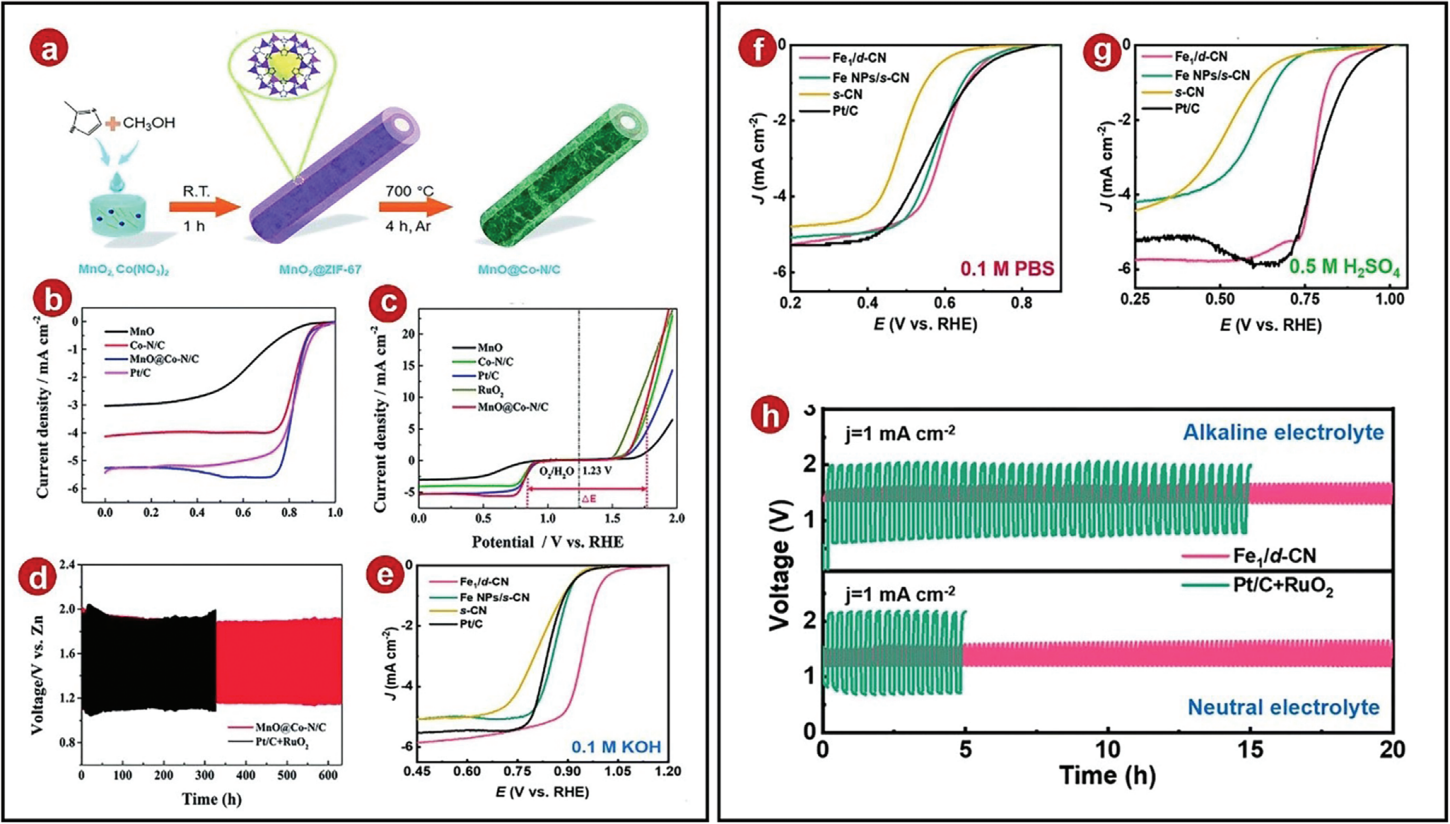
a) Preparation process diagram of MnO@Co–N/C nanowires. b) ORR polarization curves of several catalysts in 0.1 m KOH electrolytes at 1600 rpm. c) LSV curves representing the ORR and OER of several catalysts. d) Cycles of discharge–charge at 5 mA cm^−2^. e) ORR LSV curves in 0.1 m KOH media. f) ORR LSV curves in 0.1 m PBS media. g) ORR LSV curves in 0.5 m H_2_SO_4_ media. h) In flexible quasi‐solid‐state alkaline (top side) and neutral (bottom side) Zn–air batteries, stability tests were conducted using Fe_1_/d‐CN and Pt/C + RuO_2_ catalysts as the air cathode. a–d) Reproduced with permission.^[^ ^76^ ^]^ Copyright 2018, The Royal Society of Chemistry. e–h) Reproduced with permission.^[^ ^77^ ^]^ Copyright 2021, The Royal Society of Chemistry.

Traditional Zn–air batteries have low kinetic rates of ORR and OER, which have always been a big challenge for researchers. So far, adding catalyst is a good solution. Chen et al. chose ultrathin MnO_2_ hollow nanowires as templates, and successfully fabricated porous MnO@Co–N/C nanomaterials by hydrothermal method, thermal cracking, and other steps (**Figure** **9**a).^[^ ^76^ ^]^ After testing, they found that the limiting current density of MnO@Co–N/C was the highest, which was 5.25 mA cm^−2^. It exceeded the levels of other comparative samples (Figure 9b), that demonstrated the excellent catalytic performance of the MOFs composite sample for ORR. In the test of the catalytic performance of OER, the speed was adjusted to 1600 rpm, and MnO@Co–N/C reached a current density of 10 mA cm^−2^ at 1.76 V, indicating that the MOFs composite sample has an excellent catalytic activity effect on OER (Figure 9c). After that, Chen et al. assembled a Zn–air battery and applied the MnO@Co–N/C catalyst to it and then tested it for cycling. They found that at a current density of 5 mA cm^−2^, the Zn–air battery, which was composited with MOFs can be cycled stably for 1900 times with a constant voltage and no degradation (Figure 9d), all of which demonstrated the potential of MnO@Co–N/C catalysts for solid‐state Zn–air batteries.

Considering the excellent performance of the laminate structure, Zhao et al. effectively created the catalyst Fe_1_/d‐CN (defect‐rich single Fe site catalyst loaded on a hierarchical porous CN support), which had a single iron site and a lot of defects, utilizing the bilayer ZIF‐8 structure.^[^ ^77^ ^]^ In testing its catalytic performance, they discovered that the Fe_1_/d‐CN catalyst had a very high current density at 0.9 V, reaching 22.7 mA cm^−1^ under alkaline conditions (0.1 m KOH), which was dozens of times than that of other commercial batteries. Then they tested its half‐wave potential with LSV, and found the results were better than other commercial batteries (Figure 9e). In addition, in acidic and neutral environments, the ORR activity of Fe_1_/d‐CN catalyst was found to be higher. Compared with other comparative samples (Figure 9f,g), it showed that the catalyst has a wide range of applicability. Subsequently, a quasi‐solid‐state Zn–air battery combined with Fe_1_/d‐CN catalyst was assembled and it was found that under the action of the GPEs of PVA‐KOH, Fe_1_/d‐CN catalyst showed more excellent electrochemical performance: the battery was continuously charged and discharged for 20 h, then the Fe_1_/d‐CN‐based Zn–air battery had a lower charging voltage and a higher discharging platform (Figure 9h), which indicated that this battery had a good cycle performance.

**Figure 10:**
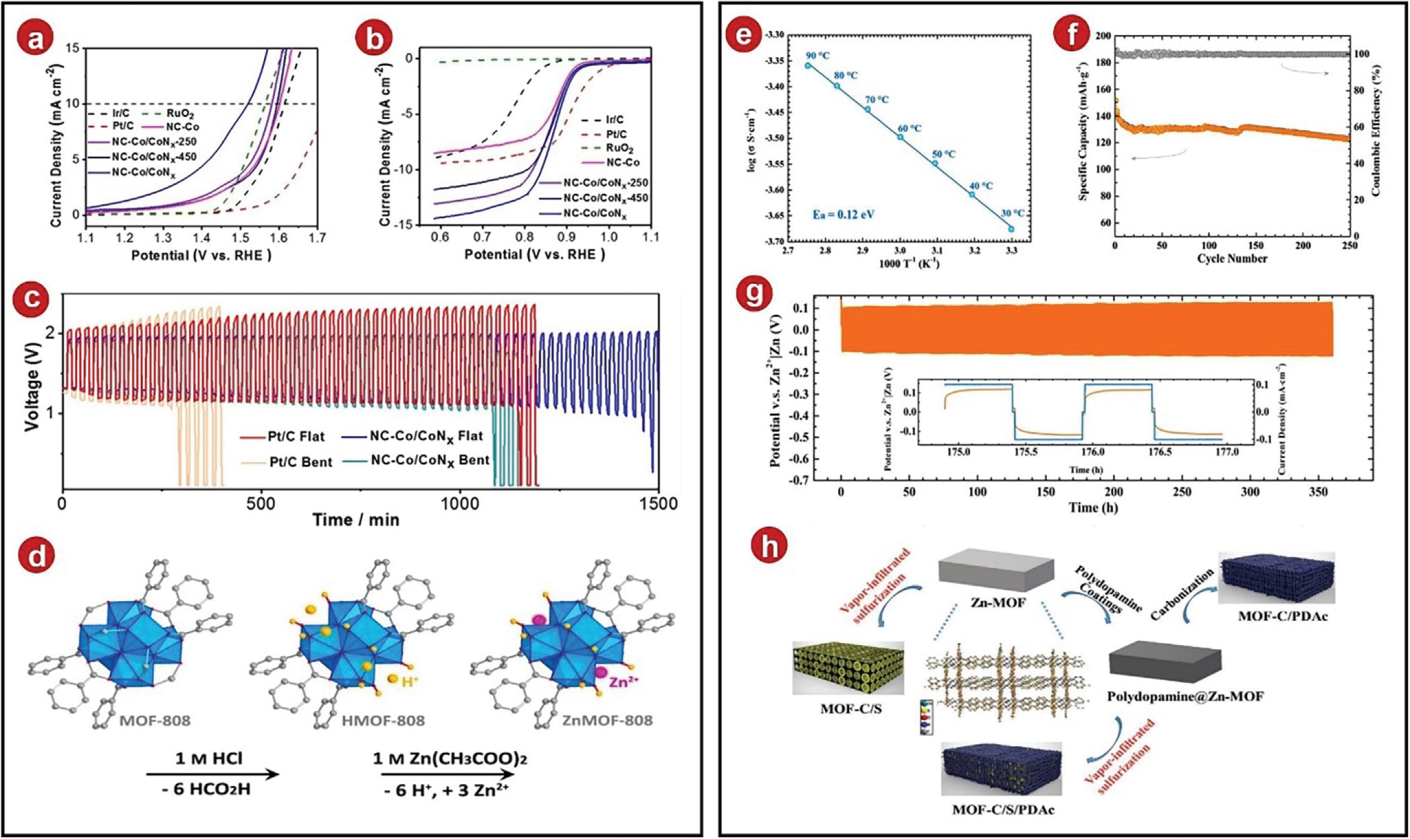
a) At 1600 rpm, the oxygen evolution polarization curves. b) At 1600 rpm, oxygen reduction polarization curves. c) Cycle stability of Pt/C and NC‐Co/CoN*_x_* cathode‐based SSBs in flat and bent states. d) Schematic diagram of postsynthesis modification. e) Ionic conductivities of WZM SSE from 30 to 90 °C were shown on an Arrhenius plot. f) Cycle performance at 0.2 A g^−1^. g) Performance of the Zn symmetric cell for Zn plating/stripping at 0.1 mA cm^−2^. h) Schematic diagram of the synthesis of Zn‐MOFs, MOFs‐C/S, MOFs‐C/PDAc, and MOFs‐C/S/PDAc composites. a–c) Reproduced with permission.^[^ ^78^ ^]^ Copyright 2019, Elsevier. d–g) Reproduced with permission.^[^ ^79^ ^]^ Copyright 2019, Elsevier.

Later, Guan et al. reported hybrid cobalt/cobalt nitride nanoparticle‐decorated nitrogen‐doped carbon (NC‐Co/CoN*_x_*) nanoarrays and applied them to Zn–air battery.^[^ ^78^ ^]^ Under the catalysis of NC‐Co/CoN*_x_* composite, the ORR and OER properties had been slightly improved compared with other contrasting samples (**Figure** **10**a,b), indicating that after the composites of C and N, the MOFs material had better catalytic performance. After assembling a solid‐state Zn–air battery containing NC‐Co/CoN*_x_*, the Zn–air battery had a higher discharge voltage and a lower charging voltage in the environment of gel electrolytes and NC‐Co/CoN*_x_* as a catalyst, and its dropout voltage became smaller, indicating that the battery had better reversibility. In addition, the small interface resistance of the gel electrolytes and the MOFs composite material was used to achieve a stable cycle of the battery, whether it was a continuous planar or curved charge–discharge cycle, it had a stable operation result (Figure 10c).

Zn‐ion battery has the advantages of low cost and good safety, but it also faces the Zn anode deposition in solution to form dendrites, affecting the practical application of the battery. As a response, a crystalline SSE containing a single‐ion Zn^2+^ was reported by Wang et al. (Figure 10d).^[^ ^79^ ^]^ In the test, the ionic conductivity of common ZnMOFs‐808 ([Zr_6_O_4_(OH)_4_(OH)_12_Zn_3_(BTC)_2_]) was not found to be excellent at room temperature, but it absorbed a certain amount of moisture in a humid environment, forming a special SSEs water@ZnMOFs‐808 (WZM), while WZM had a conductivity of up to 2.1 × 10^−4^ S cm^−1^ at 30 °C (Figure 10e) and a very small activation energy. Subsequently, the compatibility of WZM with the Zn anode was tested and it was found that the cycle at a current density of 0.1 mA cm^−2^ was 360 h, the cyclic distribution of each voltage was uniform and smooth (Figure 10g), which also showed that the contact interface between the electrolytes and the anode had been improved, avoiding the deposition of Zn dendrites. After that, the VS_2_/Zn battery was also assembled. The SSEs formed a two‐layer structure, ensuring its good mechanical stability. Charged and discharged at 0.2 Ah g^−1^, the test found that its coulomb efficiency was still 99.7% after 250 cycles (Figure 10f).

### 4.2 Na‐Based Batteries

#### 4.2.1 Working Mechanism

The mechanism of Na batteries is basically consistent with lithium batteries, therefore, traditional Na batteries also have safety problems due to the use of liquid electrolytes, such as the instability of liquid electrolytes and volatilization, generating unstable sodium dendrites.^[^ ^80^ ^]^ Consequently, the usage of SSEs is a trend in the development of sodium batteries.^[^ ^81^ ^]^ In addition to constructing ion channels that facilitate Na^+^ transport and restrict the movement of bulky anions, MOFs can be used to store and screen different ions by virtue of their own framework structures. MOFs can be effectively used in Na‐based batteries by modulating their different structural morphologies, such as pancake type, which can load more electrolytes, and red cell type, which can transport oxygen more easily and has excellent resistance to stress, etc. MOFs are also very easy to load functional groups with special effects. For example, they have strong electronegativity, increase the density of nearby electron clouds, and increase the repulsion of multielectron substances. This reduces the diffusion potential of Na^+^ and increases the migration rate of Na^+^. These functional groups also have a very important impact on improving the performance of Na‐based batteries.

#### 4.2.2 Application

**Figure 11:**
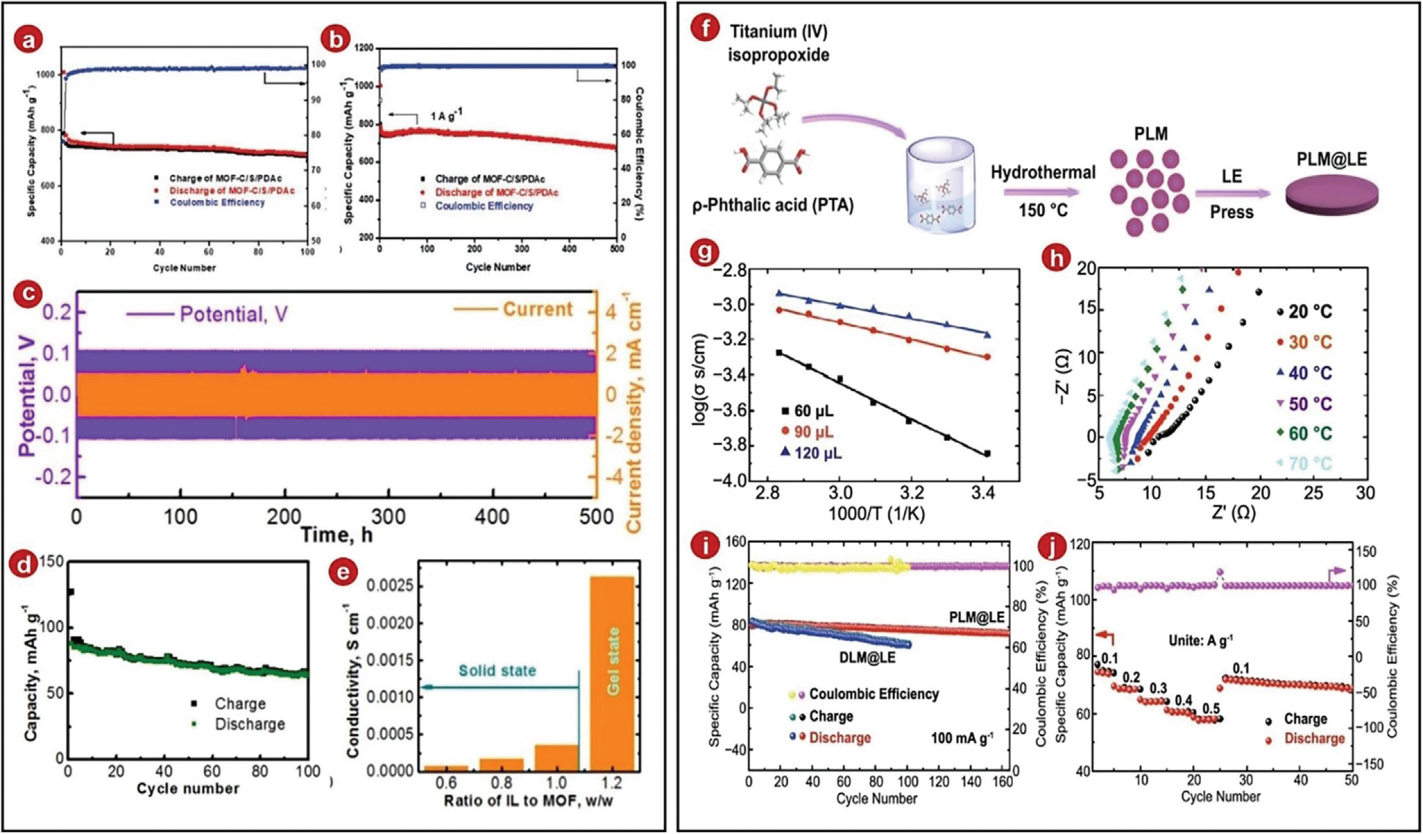
a) Performance across a cycle for the MOFs‐C/S/PDAc electrode. b) The MOFs‐C/S/PDAc electrode was cycled repeatedly at 1 A g^−1^. c) Long‐term charge–discharge polarization voltage and current profiles of the symmetric Na–Na cell. d) Charge–discharge capabilities as a function of the Na–Na_3_Ni_1.5_TeO_6_ cell cycle number at a C/10 cycling rate. e) Summary of the ionic conductivity. f) Schematic diagram of the synthesis of PLM@LE. g) PLM@LE ionic conductivity Arrhenius graphs for various LE contents. h) EIS of PLM@LE at temperatures from 20 to 70 °C. i) Cycling performance of Na_0.44_MnO_2_‐Na and Na_0.44_MnO_2_‐Na SSBs at 100 mA g^−1^. j) Rate performance of Na_0.44_MnO_2_‐Na. a–b) Reproduced with permission.^[^ ^82^ ^]^ Copyright 2020, Wiley‐VCH. c–e) Reproduced with permission.^[^ ^83^ ^]^ Copyright 2021, American Chemical Society. f–j) Reproduced with permission.^[^ ^84^ ^]^ Copyright 2021, Springer.

Na–S batteries have a very high theoretical capacity in sodium‐based batteries, so they have a very promising research prospect. However, due to the poor conductivity of sulfur and the slow reaction rate, there are many shortcomings in application. In this way, an MOFs‐derived S, N‐doped porous carbon host was designed and applied in Na–S batteries by Xiao et al., improving the electrochemical properties of the battery (Figure 10h).^[^ ^82^ ^]^ The electrode had high cycle stability under the action of MOFs composites, After cycling 100 times at 0.1 A g^−1^, it still had 691 mAh g^−1^ (**Figure** **11**a), 500 cycles at 1 A g^−1^, and 90% capacitance (Figure 11b). In addition, it had excellent results at different current densities. The reason was that because the SSEs were combined with sulfur to form covalent sulfur, the shuttle effect as prevented and its stability was greatly improved. Additionally, this demonstrated the potential use of MOFs‐derived composites in Na batteries.

Recently, Yu et al. designed and synthesized a quasi‐solid state sodium battery containing MOFs.^[^ ^83^ ^]^ They compounded sodium‐based ionic liquid laden sulfonated University of Oslo‐66 (UIOSNa) with sodium IL to form Na‐IL/UIOSNa. The ionic conductivity of the prepared GPEs was controlled by controlling the content ratio of MOFs to IL. When MOFs: IL was 1:1, the conductivity of Na^+^ was the highest, reached 3.6 × 10^−4^ S cm^−1^ (Figure 11e), the result was much larger than that of MOFs materials without IL composite. Later, Yu et al. assembled a sodium symmetric battery with Na‐IL/UIOSNa electrolytes, then they charged and discharged the battery at 1.0 mA cm^−2^. According to the results, it was found that the stable cycle time of the battery exceeded 500 h (Figure 11c), while the exception occurred after the traditional sodium battery was cycled for only 100 h, which also showed that the GPEs effectively inhibited the formation of sodium dendrites. In addition, Yu et al. also assembled a Na‐Na_3_Ni_1.5_TeO_6_ battery, then they found that the quasi‐solid battery can operate efficiently and stably as long as it was at a reasonable C rate (C/10). After 100 cycles, its capacity retention rate can reach 76.5% of the original, the coulomb efficiency was basically higher than 99% (Figure 11d), which proved the excellent application prospect of Na‐IL/UIOSNa in sodium batteries.

In addition, it is also a good plan to regulate the MOFs from its own morphology. According to a red blood cell model, a pancake‐shaped MOFs framework was created and it was coupled with the original liquid electrolytes to create the new SSEs, pancake‐like MIL‐125@liquid electrolyte (PLM@LE) (Figure 11f).^[^ ^84^ ^]^ After that, relevant tests were carried out in the symmetrical battery composed of stainless steel. According to the result, they realized that the content of LE was controlled at 90 µL, PLM@LE has the lowest activation energy (Figure 11g). On this basis, at 20 °C the ionic conductivity of PLM@LE was found to be 6.60 × 10^−4^ (Figure 11h), these results were better than the comparison sample. In the experiment, it was found that the unique pore size and group of PLM can effectively improve the sodium ion migration number. Later, Zhang et al. assembled Na_0.44_MnO_2_‐Na battery. After cycling for 160 times under the current density of 100 mA g^−1^, its specific capacity still had ≈90% of the original (Figure 11i), and the discharge capacity of the battery had good results after adjusting the current density of different sizes (Figure 11j), which further illustrated that PLM@LE high efficiency and stability of electrolytes in solid‐state sodium battery.

## 5 Conclusions and Outlook

Performance of MOFs in various SSEs and the application of MOFs materials as SSEs in various batteries (Li‐ion batteries, Li–S batteries, Li–O_2_ batteries, Zn‐based batteries, Na‐based batteries) are also reviewed in this review. The results show that MOFs materials have high potential for improving battery electrochemical properties and promoting battery cycle stability by virtue of their advantages such as high porosity, high specific surface area, excellent dynamics, and diversified structures, whether as 3D skeletons, complexes of SSEs or derivatives. However, the application of MOFs in SSEs is still in its infancy, and although many exciting results have been found so far, there are still many challenges in this research direction. Here, some of the problems and challenges in the field are presented.

1) The mode of lithium‐ion transport in electrolytes has not been fully explained, the mechanism of the reduction of high interfacial resistance and the improvement of Li^+^ transport in some solid–solid contact interfaces has not been thoroughly studied, the conductivity of many MOFs and its derivatives materials is not excellent, the higher internal charge transfer resistance will also increase the heat dissipation of the battery, affecting the cycle stability of the battery.

2) Regarding the pore size, morphology, crystal size and structure, different metal sites of MOFs materials, their mechanism for ion conduction and improvement of electrochemical properties needs to be further studied. Different anionic sites and lithium metal interaction can improve the ionic conductivity of SSEs, but it is not clear that these lithium metal sites and anion mechanism of different kinds of MOFs, such as some have special morphology or contain other particles of MOFs, their impact on the electrochemical performance of the SSEs and mechanism also needs to be further explored.

3) MOFs materials have many other hidden functions, such as the ability to take advantage of the large specific surface area and high porosity properties of MOFs to absorb contaminants produced by some batteries during operation. Many of these features have great commercial value and need to be further developed.

4) Many MOFs materials in the application of SSEs, involving expensive lithium‐ion, ligands, and complex synthesis methods, their preparation will take a lot of time and money, seriously affecting the production costs of commercial applications and limiting the application of MOFs in SSEs to a certain extent.

Although there are many issues and challenges in MOFs composites, to solve them, some solutions are given.

1) MOFs can be combined with some materials with high conductivity properties, and the MOFs composite material formed can take into account the advantages of both sides and make up for their respective shortcomings, thus reducing resistance and reducing battery heat.
2) The pore size, structure, crystal size, crystallinity, etc. of MOFs materials can be fine‐tuned, and the changed properties can be compared with the changes in electrochemical properties one by one. Besides these, induction can be studied in microscopic quantum chemistry. In addition, necessary theoretical calculations and simulation experiments are also essential.
3) Analyzing some possible defects in the battery, and try to improve them from the structure and properties of the MOFs material, so that there may be new discoveries. In addition, more summary can be made to compare the electrochemical properties of different kinds of MOFs‐based SSEs, and try to find out some special properties for further study.
4) Inexpensive ligands and raw materials should be selected as far as possible, simple synthesis steps (such as hydrothermal method) should be designed, and the structure of MOFs materials should be optimized to make commercial production of SSEs possible.

The applications of MOFs in SSEs have been a very important field of study, although some problems still exist in the this study, however, as the people in this area even more seriously, experimental technology progress unceasingly, more promising MOFs based materials will be excavated and MOFs future research in SSEs is bound to have more breakthroughs.

## Conflict of Interest

The authors declare no conflict of interest.
